# Supplementary material for: Psychometric properties of upper limb kinematics during functional tasks in children and adolescents with dyskinetic cerebral palsy
Source: PLoS One. 2022 Sep 23;17(9):e0266294. doi: 10.1371/journal.pone.0266294 (PMC9506636; doi:10.1371/journal.pone.0266294)
Supplement: S1 Table — TD = typically developing; DCP = dyskinetic cerebral palsy; MACS = manual ability classification system; M = male; F = female. (DOCX) [file pone.0266294.s001.docx]

| Table S1: Participant characteristics | | | | | |
| --- | --- | --- | --- | --- | --- |
| Participant | **Age** | **SEX** | **MACS level** | **L/R handed** | **Measured side** |
| DCP1 | 12.2y | M | 3 | R | L |
| DCP2 | 23.9y | F | 1 | R | L |
| DCP3 | 23.9y | F | 1 | R | R |
| DCP4 | 18.5y | F | 2 | L | L |
| DCP5 | 25.4y | M | 3 | L | R |
| DCP6 | 16.2y | M | 2 | R | L |
| DCP7 | 16.2y | M | 2 | R | R |
| DCP8 | 13.5y | M | 2 | L | R |
| DCP9 | 19.4y | F | 2 | R | L |
| DCP10 | 16.9y | M | 3 | R | L |
| DCP11 | 16.9y | M | 3 | R | R |
| DCP12 | 11.9y | M | 2 | R | L |
| DCP13 | 11.9y | M | 2 | R | R |
| DCP14 | 13.6y | F | 3 | R | L |
| DCP15 | 21.6y | F | 3 | R | L |
| DCP16 | 8.6y | M | 2 | L | R |
| DCP17 | 8.8y | F | 2 | R | L |
| DCP18 | 22.6y | M | 2 | L | R |
| DCP19 | 9.6y | M | 2 | R | L |
| DCP20 | 19.2y | F | 3 | R | L |
|  |  |  |  |  |  |
| TD1 | 9.4y | M | / | R | L |
| TD2 | 19.9y | M | / | R | L |
| TD3 | 22.2y | F | / | R | L |
| TD4 | 15.1y | F | / | R | L |
| TD5 | 14.1y | F | / | L | R |
| TD6 | 17.0y | F | / | R | L |
| TD7 | 18.2y | M | / | R | L |
| TD8 | 21.2y | F | / | R | L |
| TD9 | 21.5y | F | / | R | L |
| TD10 | 21.4y | F | / | R | L |
| TD11 | 13.5y | M | / | L | R |
| TD12 | 19.5y | F | / | R | L |
| TD13 | 15.0y | F | / | R | L |
| TD14 | 12.1y | M | / | R | L |
| TD15 | 21.2y | F | / | R | L |
| TD16 | 13.3y | M | / | R | L |
| TD17 | 14.4y | F | / | L | R |
| TD18 | 12.9y | F | / | R | L |
| TD19 | 16.9y | M | / | R | L |
| TD20 | 24.2y | F | / | R | L |
| TD = typically developing; DCP = dyskinetic cerebral palsy; MACS = manual ability classification system; M = male; F = female | | | | | |
